# Homes for Invalids

**Published:** 1876-06

**Authors:** 


### *HOMES FOR INVALIDS.*

The most doleful and unfortunate place that an invalid can get into is one of those extensively advertised "homes." Such places are generally in charge of some "strong minded woman," or some man of but one idea, and that of the most whimsical and absurd kind. About a year since a young friend of ours, a bright, promising young man, being somewhat worn down with study and confinement to a school-room, sought rest and recreation at a "home" where he was starved and drenched with cold water during four weeks, and then came home weighing twenty pounds less than when he went away, and a hopeless consumptive. He survived but a few months after his return. Had he not fallen into the "home" sna e he would probably have recovered under the kind care and attention of his family. In our judgment consumptives usually make a mistake to exchange a comfortable home—wherever located—for the discomforts certain to be experienced at most popular places of resort for invalids. We are at a loss to discover what such places offer more than can be found in any healthy locality. We advise persons with weak lungs to find employment that will keep them as much as possible in the open air and sunshine ; to throw back their shoulders so that the lungs will not be compressed, and at each inhalation to admit as much air to the lungs as is possible without inconvenience or fatigue ; to wear thick flannels summer and winter ; to keep the feet dry and warm ; to take at least eight hours sleep and rest out of every twenty-four hours, and to live generously. The system can no more maintain its vigor and health, and resist the march of disease without a sufficiency of strong, wholesome food, than a steam engine can be run without fuel.

